# Drug-utilisation profiles and COVID-19

**DOI:** 10.1038/s41598-021-88398-y

**Published:** 2021-04-26

**Authors:** Valentina Orlando, Enrico Coscioni, Ilaria Guarino, Sara Mucherino, Alessandro Perrella, Ugo Trama, Giuseppe Limongelli, Enrica Menditto

**Affiliations:** 1 CIRFF, Center of Drug Utilisation and Pharmacoeconomics, University of Naples Federico II, 80131 Naples, Italy; 2 Division of Cardiac Surgery, AOU San Giovanni di Dio E Ruggi d’Aragona, 84131 Salerno, Italy; 3 Infectious Disease of Healthcare Direction, AORN Antonio Cardarelli, 80131 Naples, Italy; 4 Regional Pharmaceutical Unit, Campania Region, 80143 Naples, Italy; 5 Department of Translational Medical Science, University of Campania Luigi Vanvitelli, Monaldi Hospital, 80131 Naples, Italy; 6 Department of Pharmacy, Center of Drug Utilisation and Pharmacoeconomics, University of Naples Federico II, 80131 Naples, Italy

**Keywords:** Epidemiology, Outcomes research, Health policy, Infectious diseases

## Abstract

Coronavirus disease 2019 (COVID-19) has substantially challenged healthcare systems worldwide. By investigating population characteristics and prescribing profiles, it is possible to generate hypotheses about the associations between specific drug-utilisation profiles and susceptibility to COVID-19 infection. A retrospective drug-utilisation study was carried out using routinely collected information from a healthcare database in Campania (Southern Italy). We aimed to discover the prevalence of drug utilisation (monotherapy and polytherapy) in COVID-19 versus non-COVID-19 patients in Campania (~ 6 million inhabitants). The study cohort comprised 1532 individuals who tested positive for COVID-19. Drugs were grouped according to the Anatomical Therapeutic Chemical (ATC) classification system. We noted higher prevalence rates of the use of drugs in the ATC categories C01, B01 and M04, which was probably linked to related comorbidities (i.e., cardiovascular and metabolic). Nevertheless, the prevalence of the use of drugs acting on the renin-angiotensin system, such as antihypertensive drugs, was not higher in COVID-19 patients than in non-COVID-19 patients after adjustments for age and sex. These results highlight the need for further case–control studies to define the effects of medications and comorbidities on susceptibility to and associated mortality from COVID-19.

## Introduction

As of 24 April 2020, there has been ~ 3,000,000 coronavirus disease 2019 (COVID-19) cases and > 200,000 associated deaths worldwide^1^. COVID-19 is very contagious and has a wide spectrum of presentations. COVID-19 symptoms can range from no symptoms to severe illness, and the disease includes three phases (i.e., viral infection, pulmonary, hyperinflammation/systemic phases)^2^. Ageing and underlying diseases (e.g., heart disease, diabetes mellitus) have been reported to be risk factors for adverse outcomes, and male sex and a genetic predisposition to infection are under investigation as potential contributors^3–7^. Moreover, initial reports suggested a potential pro-infective effect of drugs. Two classes of drugs that have been implicated are angiotensin-converting enzyme inhibitors (ACEIs) and angiotensin II receptor blockers (ARBs). These effects may be due to the interaction between the virus that causes COVID-19, severe acute respiratory syndrome coronavirus 2 (SARS-CoV-2), and ACE-2 receptors in the lungs, though this theory is controversial^8–12^.

There is a lack of data on drug use (monotherapy and polytherapy) in COVID-19 patients. The main aims of this study were to (1) discover the prevalence of drug utilisation (monotherapy and polytherapy) in COVID-19 versus non-COVID-19 patients in Campania, southern Italy and (2) ascertain the epidemiology and profiles of affected patients in relation to drug utilisation.

## Methods

### Study design

A retrospective drug-utilisation study was carried out using routinely collected information from healthcare databases in Campania. The Campania Region Database (CaReDB) includes information on patient demographics and the electronic records of outpatient pharmacy dispensing for ~ 6 million residents, comprising a well-defined population in Italy (~ 10% of the population of Italy). CaReDB is complete and includes data that has been validated in previous drug-utilisation studies^13–20^. The characteristics of CaReDB are described in Supplementary Table S1.

At the beginning of the COVID-19 epidemic, a surveillance system was implemented to collect the data of all cases identified by reverse transcription-polymerase chain reaction (RT-PCR) testing for SARS-CoV-2. These archives are linked together by a unique anonymous identifier that is encrypted to protect patient privacy. Our research protocol adhered to the tenets of the Declaration of Helsinki 1975 and its later amendments. Permission to use anonymised data for this study was granted to the researchers of the Centro di Ricerca in Farmacoeconomia e Farmacoutilizzazione (CIRFF) by the governance board of Unità del Farmaco della Regione Campania. The research did not involve a clinical study, and all patients’ data were fully anonymised and were analysed retrospectively. For this type of study, formal consent was not required according current national established by the Italian Medicines Agency, and according to the Italian Data Protection Authority, neither ethical committee approval nor informed consent was required for our study^21^.

### Study population

People who had been dispensed medication according to CaReDB during 2019 were included in the study cohort. From regional surveillance system data, we obtained the information of patients with confirmed COVID-19 from the beginning of the epidemic (26 February 2020) to 30 March 2020 who were linked to the population identified in CaReDB. For the purposes of our investigation, the study population diagnosed with SARS-CoV-2 infection on or before the date of analysis was referred to as the ‘COVID-19 group’ (C19G). The remaining individuals were used as a comparator group in the analysis and were referred to as the ‘general population group’ (GPG).

### Patient characteristics

The study population was categorised by sex and subdivided into four age groups: 0–39; 40–59; 60–79; and ≥ 80 years. The number of drug prescriptions, prevalence of drug use and polypharmacy regimens (classified as ‘no-polypharmacy’; ‘polypharmacy’; and ‘excessive polypharmacy’) were ascertained in 2019. Drugs were grouped according to the Anatomical Therapeutic Chemical (ATC) classification system. ATC II and ATC IV codes with a prevalence ≥ 3% in the C19G were included in the analysis.

### Outcome

The drug-utilisation profile was evaluated as the prevalence of drug use. Drug use prevalence was estimated as the number of individuals dispensed ≥ 1 drug prescription per 100 inhabitants in 2019. The prevalence of drug use was evaluated in the C19G and GPG. Prevalence was stratified by age group and sex. Prevalence was probably influenced by the heterogeneous demographic distribution among the age groups, so we conducted direct standardisation.

### Statistical methods

The baseline characteristics of the study population were analysed using descriptive statistics. Quantitative variables are described as means ± standard deviations. Categorical variables are described as counts and percentages. The chi-square test and t-test were performed to determine the difference between the C19G and GPG in terms of sex and age. A *P*-value of < 0.05 was considered significant. Crude and age-adjusted prevalence rates were calculated. Differences in the prevalence between the C19G and GPG are expressed as risk ratios (RRs) adjusted for sex and age with 95% confidence intervals (CIs). Standardisation was performed using a direct method whereby the Italian population up to 1 January 2019 was used as the standard population (available on the Demo Istat website^22^).

$${\text{Direct}}\,{\text{standardised}}\,{\text{rate}}\, = \,\frac{{\sum\nolimits_{i = 1}^{m} {w_{i} \cdot T_{i} } }}{{\sum\nolimits_{i = 1}^{m} {w_{i} } }} \cdot k$$

where (*T*_*i*_ = *n*_*i*_/*n*) = rate in stratum ‘*i*’ of the study population; *n*_*i*_ = number of cases in stratum ‘*i*’ of the study population; *N* = size of the study population in stratum ‘*i*’; *w*_*i*_ = size of stratum ‘*i*’ of the reference population; *m* = number of considered strata; *k* = multiplicative constant.

The age-adjusted RRs and 95% CIs were computed using standard methods. Data management was performed with SQL server v2018 (Microsoft, Redmond, WA, USA). Analyses were carried out with SPSS v17.1 (IBM, Armonk, NY, USA).

## Results

### C19G characteristics

A total of 1,532 individuals in Campania who tested positive for COVID-19 on 30 March 2020 were identified. Of these, 926 (60.4%) were males, and the median age of the entire sample was 55 ± 19 years. Among the C19G patients, 20.8% were aged 0–39 years, 36.1% were aged 40–59 years, 33.6% were aged 60–79 years, and 9.5% were aged > 80 years. The percentage of males was higher than that of females in all age groups except the > 80 years age group (43.8% males). Differences in age and the sex ratio between the C19G and GPG were statistically significant (*p*-value < 0.001).

The prevalence of drug use among the C19G was 74.5% and increased with age, reaching 93.8% in those aged > 80 years. The median number of prescriptions per patient (overall: 16 [interquartile range, IQR]: 5–42) ranged from 3 (IQR, 1–6) among people aged 0–39 years to 51 (IQR, 29–71) among individuals aged > 80 years.

Half of the COVID-19 patients aged 0–39 years had no exposure to any medication, whereas 45.5% of the COVID-19 patients were prescribed ≤ 4 medications, and 4.1% had polypharmacy regimens (5–9 drugs). The percentage of participants receiving polypharmacy increased with increasing age, at 18.3% in those aged 40–59 years and 34.8% in those aged 60–79 years; moreover, ~ 80% of participants aged > 80 years were prescribed polypharmacy or excessive polypharmacy (≥ 10 drugs) regimens. The C19G characteristics are shown in Table 1.

**Table 1:** Characteristics of the COVID-19 population.

	Overall 1532	Age groups N (%)			
		0–39 years 319 (20.8)	40–59 years 553 (36.1)	60–79 years 514 (33.6)	≥ 80 years 146 (9.5)
**Sex N (%)**					
Male	926 (60.4)	189 (59.2)	335 (60.6)	338 (65.8)	64 (43.8)
Female	606 (39.6)	130 (40.8)	218 (39.4)	176 (34.2)	82 (56.2)
Mean age ± SD	55 (± 19)	27 (± 9)	51 (± 5)	68 (± 6)	85 (± 4)
Prevalence of drug use (%)	74.54	49.53	69.98	89.49	93.84
Median number of prescriptions (IQR)	16 (5–42)	3 (1–6)	9 (3–20)	28 (13–54)	51 (29–71)
**Polypharmacy group, N (%)**					
0 drugs	387 (25.5)	161 (50.5)	163 (29.5)	54 (10.5)	9 (6.2)
No polypharmacy (1–4 drugs)	600 (39.2)	145 (45.5)	264 (47.7)	168 (32.7)	23 (15.8)
Polypharmacy (5–9 drugs)	351 (22.9)	13 (4.1)	101 (18.3)	179 (34.8)	58 (39.7)
Excessive polypharmacy (≥ 10 drugs)	194 (12.7)	–	25 (4.5)	113 (22.0)	56 (38.4)

### Drug-utilisation profiles of the C19G

**Figure 1:**
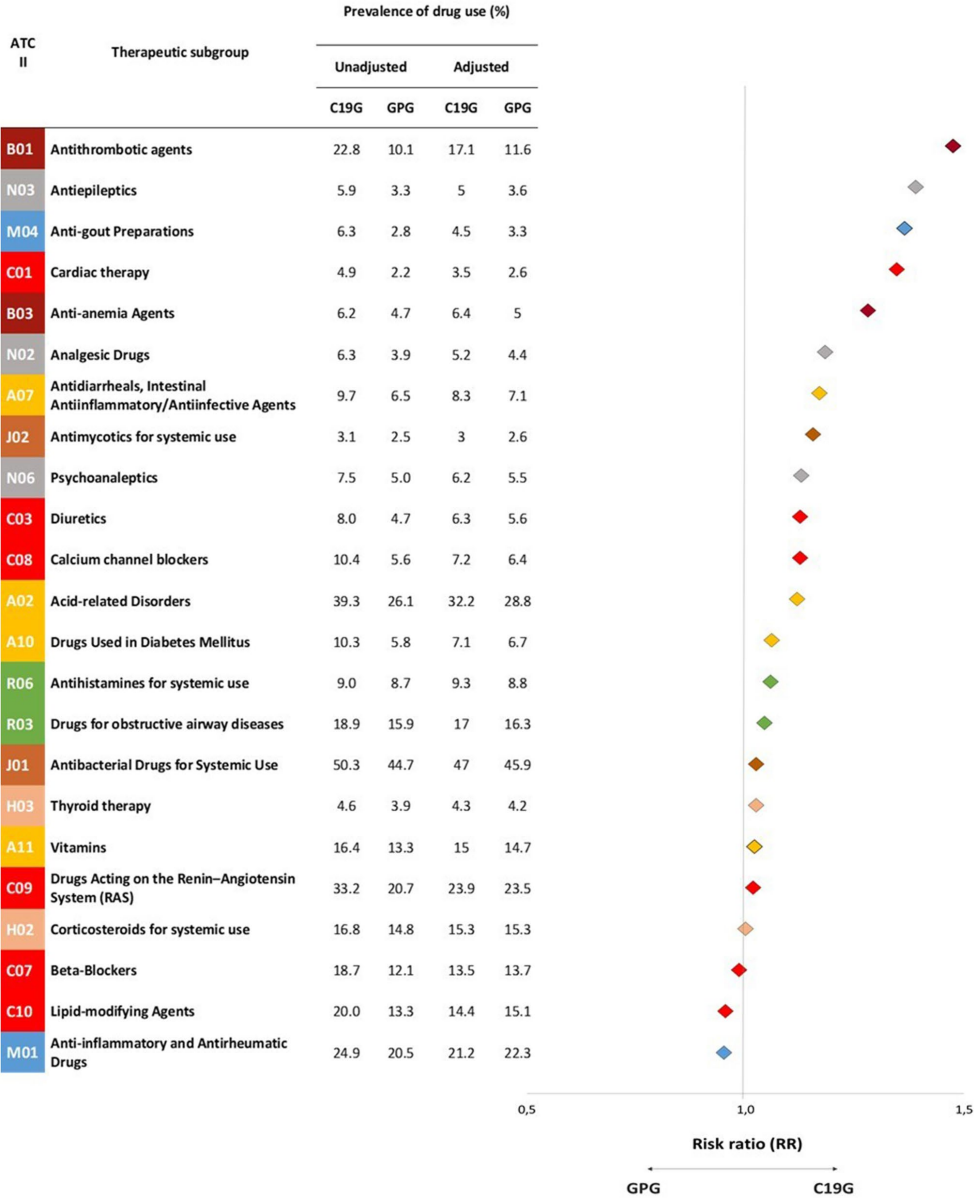
Differences in prevalence of drug use between the C19G and GPG according to Therapeutic Group (ATC II). *C19G* COVID-19 group; *GPG* general population group.

Twenty-three pharmacological ATC II groups and 39 ATC IV groups had a prevalence > 3% in the C19G. The highest unadjusted and adjusted prevalence rates of drug use in the ATC II groups were observed for drug categories J01, A02, C09, M01, B01 and R03 in the C19G and GPG (Fig. 1).

Crude differences (at least ± 20% in the overall prevalence of drug use between the C19G and GPG) were found in all 23 pharmacological ATC II groups and in 30 of 39 ATC IV groups included in the analysis (Fig. 1, Table 2). After adjustment, differences remained in six ATC II groups and eight ATC IV groups. With respect to drugs acting on the renin–angiotensin system (RAS) (C09), including beta-blockers (C07), antibacterial drugs for systemic use (J01) and anti-inflammatory and antirheumatic drugs (M01), the differences disappeared after adjustment. The large differences in antithrombotic agents (B01), cardiac therapy drugs (C01) and anti-epileptics (N03) diminished after adjustment, even though they were more common in the C19G than in the GPG after adjustment.

**Table 2:** Differences in prevalence of drug use between the C19G and GPG according to Chemical Subgroup (ATC IV).

ATC IV	Chemical subgroup	Prevalence of drug use (%)				Adjusted RR C19G/GPG (95%CI)
		Unadjusted		Adjusted		
		C19G	GPG	C19G	GPG	
A02AD	Aluminium, calcium and magnesium	4.6	3.1	3.7	3.4	1.10 (1.099–1.109)
A02BC	Proton pump inhibitors	36.8	23.4	29.6	26.0	1.14 (1.136–1.140)
A02BX	Other drugs for peptic ulcers	6.9	5.1	6.1	5.5	1.10 (1.098–1.106)
A07AA	Antibiotics	7.2	5.4	6.1	5.9	1.03 (1.026–1.033)
A10BA	Biguanides	6.9	3.8	4.6	4.3	1.09 (1.083–1.092)
A11CC	Vitamin D and analogues	16.4	13.3	15.0	14.7	1.02 (1.016–1.021)
B01AB	Heparin group	5.2	2.2	4.7	2.5	1.88 (1.874–1.895)
B01AC	Platelet-aggregation inhibitors	17.2	8.1	12.2	9.4	1.29 (1.286–1.294)
B03BB	Folic acid and derivatives	4.0	2.8	3.9	3.0	1.31 (1.303–1.316)
C03CA	Sulfonamides	5.9	3.6	4.7	4.4	1.07 (1.063–1.072)
C07AB	β-blocking agents, selective	14.8	9.3	10.5	10.6	0.99 (0.988–0.994)
C08CA	Dihydropyridine derivatives	9.6	5.2	6.7	6.0	1.11 (1.105–1.113)
C09AA	ACE inhibitors	8.8	5.9	6.1	6.7	0.91 (0.902–0.909)
C09BA	ACE inhibitors and diuretics	5.0	3.2	3.6	3.7	0.97 (0.962–0.971)
C09CA	Angiotensin-II receptor blockers	10.2	5.7	7.4	6.5	1.13 (1.129–1.137)
C09DA	Angiotensin-II receptor blockers and diuretics	8.6	5.2	6.5	5.9	1.10 (1.099–1.107)
C10AA	HMG CoA reductase inhibitors	17.0	11.5	12.1	13.1	0.92 (0.922–0.926)
H02AB	Glucocorticoids	16.8	14.8	15.3	15.3	1.00 (1.001–1.006)
H03AA	Thyroid hormones	4.2	3.6	4.0	3.8	1.05 (1.044–1.053)
J01CA	Penicillins with extended spectrum	3.7	4.0	3.4	4.1	0.83 (0.831–0.838)
J01CR	Combinations of penicillins	22.8	21.3	21.2	21.8	0.97 (0.970–0.973)
J01DD	Third-generation cephalosporins	16.8	13.4	15.5	14.1	1.10 (1.097–1.102)
J01FA	Macrolides	14.2	12.7	13.8	12.9	1.07 (1.067–1.072)
J01MA	Fluoroquinolones	14.6	10.1	12.0	11.0	1.09 (1.082–1.088)
J01XX	Other antibacterials	5.6	4.5	5.4	4.9	1.11 (1.101–1.110)
J02AC	Antimycotic for systemic use	3.1	2.5	3.0	2.6	1.17 (1.160–1.172)
M01AB	Acetic acid derivatives	10.8	8.3	9.0	9.1	1.00 (0.994–1.000)
M01AE	Propionic acid derivatives	12.3	10.8	10.7	11.7	0.92 (0.913–0.918)
M01AH	Coxibs	4.1	3.2	3.3	3.5	0.94 (0.938–0.947)
M01AX	Other anti-inflammatory and antirheumatic agents, non-steroidal anti-inflammatory drugs	4.0	4.3	3.0	4.7	0.63 (0.632–0.637)
M04AA	Preparations inhibiting uric acid	5.9	2.7	4.2	3.2	1.29 (1.286–1.299)
N03AX	Other antiepileptics	4.0	2.3	3.4	2.6	1.30 (1.294–1.308)
N06AB	Selective serotonin reuptake inhibitors	3.9	3.4	3.3	3.8	0.86 (0.853–0.860)
N06AX	Other antidepressants	3.8	1.7	3.0	2.0	1.54 (1.531–1.550)
R03AK	Adrenergics in combination with corticosteroids	5.9	4.2	4.8	4.5	1.06 (1.058–1.066)
R03BA	Glucocorticoids	11.2	10.4	10.7	10.3	1.03 (1.030–1.036)
R03BB	Anticholinergics	4.0	1.9	2.8	2.2	1.25 (1.241–1.256)
R06AE	Piperazine derivatives	4.8	4.5	5.0	4.6	1.10 (1.093–1.101)
R06AX	Other antihistamines for systemic use	4.6	4.6	4.8	4.7	1.02 (1.016–1.023)

*C19G* COVID-19 group; *CI* confidence interval; *GPG* general population group; *RR*, risk ratio.

### ATC A: drugs targeting the alimentary tract and metabolism

**Figure 2:**
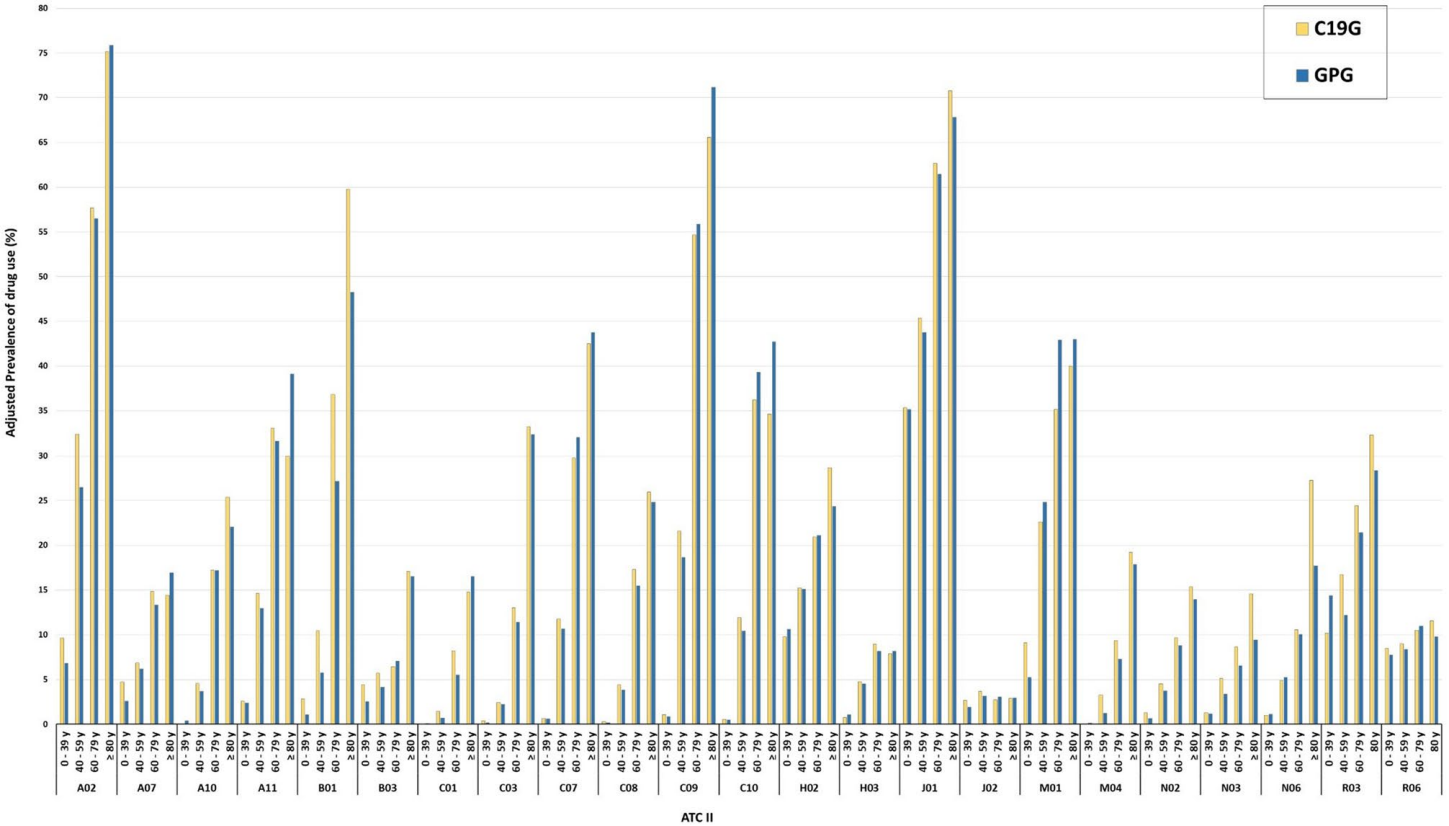
Prevalence of drug use between the C19G and GPG stratified by age group. *C19G* COVID-19 group; *GPG* general population group

Drugs for acid-related disorders (ATC II: A02) had adjusted prevalence rates of 32.2% in the C19G and 28.8% in the GPG (RR, 1.12; 95% CI, 1.116–1.120) (Fig. 1). This difference increased mainly in those aged 40–59 years (32.4% vs. 26.5%; RR, 1.22) (Fig. 2). Regarding chemical subgroups, proton pump inhibitor (ATC IV: A02BC) use had a higher prevalence in the C19G than in the GPG, mainly in those aged 0–39 years (6.8% vs. 5.2%; RR, 1.36) and 40–59 years (30.1% vs. 22.8%; RR, 1.32) (Supplementary Tables S4, S5). The difference in the prevalence of drug use for diabetes mellitus (ATC II: A10) between the C19G and GPG after adjustment was very small. With regard to ATC IV, biguanide (A10BA) use had a higher prevalence in the C19G than in the GPG, mainly in those aged ≥ 80 years (14.6% vs. 10.7%; RR, 1.36) (Supplementary Tables  S4, S5).

### ATC B: drugs targeting blood and blood-forming organs

**Figure 3:**
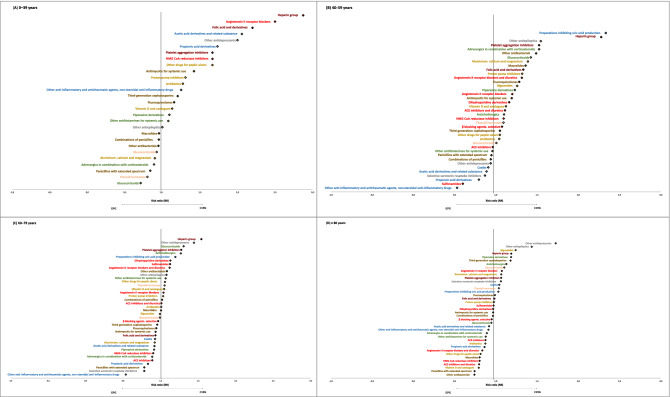
Chemical Subgroup of the C19G with the highest adjusted relative differences in prevalence stratified by age group. (**A**) Patients aged 0–39 years. (**B**) Patients aged 40–59 years. (**C**) Patients aged 60–79 years. (**D**) Patients aged > 80.

Antithrombotic agents (ATC II: B01) was the therapeutic group with the highest adjusted prevalence difference between the C19G and GPG (17.1% vs. 11.6%; RR: 1.47; 95% CI: 1.467–1.475) (Fig. 1). All age groups showed differences in adjusted prevalence rates between the C19G and GPG, with higher RRs observed in the younger age groups (Supplementary Tables S2, S3). An identical trend was observed for ATC IV. Heparin (B01AB) and platelet aggregation inhibitor (B01AC) use had higher adjusted prevalence rates in the C19G than in the GPG, with higher RRs in participants < 60 years of age (heparin: RR, 3.19 for 0–39 years and RR, 2.27 for 40–59 years; platelet aggregation inhibitors: RR, 1.94 for 0–39 years and RR, 1.52 for 40–59 years) (Fig. 3). Folic acid and derivative (B03BB) use had a higher prevalence in the C19G than in the GPG, mainly in those aged 0–39 years (3.3% vs. 1.5%; RR, 2.22) (Supplementary Tables S4, S5).

### ATC C: drugs targeting the cardiovascular system

Among drugs targeting the cardiovascular system, cardiac therapy (ATC II: C01) use had the largest differences in adjusted prevalence rates between the C19G and GPG overall and in each age group; the difference decreased with age (0–39 years: RR, 4.63; 40–59 years: RR, 2.09; 60–79 years: RR, 1.50) (Supplementary Table S3).

The other ATC II therapeutic group that pertained to the cardiovascular system did not show a relevant difference in the overall adjusted prevalence between the C19G and GPG (Fig. 1). Nevertheless, after stratification by age group, a higher RR (C19G/GPG) in people aged < 60 years was noted. In those older than 80 years, the differences disappeared or reversed, specifically for agents acting on the RAS (ATC II: C09) and lipid-modifying agents (ATC II: C10) (65.6% vs. 71.2% and 34.6% vs. 42.7% in the C19G vs. GPG, respectively) (Fig. 2).

### ATC J: anti-infectives for systemic use

Relevant differences were not observed in the overall adjusted prevalence between the C19G and GPG for the therapeutic group (ATC II) included in this drug category (Fig. 1). Nevertheless, focusing on chemical subgroups (ATC IV), among people less than 40 years of age, third-generation cephalosporin (J01DD) use had a higher prevalence in the C19G than in the GPG (11.8% vs. 9.8%; RR, 1.20). In the 40–59 years group, macrolide (J01FA) and fluoroquinolone (J01MA) use had higher prevalence rates in the C19G group than in the GPG group (16.2% vs. 11.9%, RR, 1.37; 13.1% vs. 10.2%, RR, 1.29, respectively). Among those aged > 80 years, third-generation cephalosporin (J01DD) use had a higher prevalence in the C19G than in the GPG (37.3% vs. 29.1%, RR, 1.28) (Fig. 3 and Supplementary Tables S4, S5).

With regard to anti-mycotics for systemic use (ATC IV: J02AC), a large sex difference in the overall adjusted prevalence in the C19G was noted (male RR: 1.41) (Supplementary Tables S5).

### ATC M: drugs targeting the musculoskeletal system

Regarding anti-inflammatory and antirheumatic drug (ATC II: M01) use, no significant differences were observed in the overall adjusted prevalence rates between the C10G and CPG (Fig. 1). Focusing on chemical subgroups (ATC IV), acetic acid derivative and related substance (M01AB; RR, 2.07) use and propionic acid derivative (M01AE; RR, 1.75) use had a higher prevalence rates in those aged > 40 years (Fig. 3).

Anti-gout preparation (ATC II: M04) use had adjusted prevalence rates of 4.5% in the C19G and 3.3% in the GPG (RR, 1.37; 95% CI, 1.36–1.37) (Fig. 1). A large sex difference in the overall adjusted prevalence in the C19G was observed (female RR, 1.55) (Supplementary Table S3.

Focusing on chemical subgroups (ATC IV), use of preparations inhibiting uric acid production (M04AA) had a higher prevalence in the C19G in those aged 40–59 years (2.8% vs. 1.2%; RR, 2.36) and 60–79 years (8.5% vs. 7.1%; RR, 1.21) (Supplementary Tables S4, S5).

### ATC N: drugs targeting the nervous system

Among drugs targeting the nervous system, anti-epileptic (ATC II: N03) use had the largest prevalence difference between the C19G and GPG (5.0% vs. 3.6%; RR, 1.39) (Fig. 1). For the pertaining chemical subgroup of other anti-epileptics (ATC VI: N03AX), the RR in COVID-19 patients increased with age, reaching its highest value in those aged > 80 years (11.7% vs. 7.2%; RR, 1.62) (Supplementary Tables S4, S5). Psychoanaleptic (ATC II: N06) use had adjusted prevalence rates of 6.2% in the C19G and 5.5% in the GPG (RR, 1.12; 95% CI, 1.114–1.122) (Fig. 1).

Focusing on chemical subgroups, other antidepressant (ATC IV: N06AX) use had a high prevalence in COVID-19 patients in all age groups except for those aged 40–59 years (Fig. 3).

Sex differences were observed for analgesic drug (N02) use (male RR, 1.41), other anti-epileptic (N03AX) use (female RR, 1.55) and selective serotonin reuptake inhibitor (N06AB) use (male RR, 0.67) (Supplementary Tables S3, S5).

### ATC R: drugs targeting the respiratory system

Marked differences in the prevalence of therapeutic group (ATC II) use were not observed between the C19G and GPG (Fig. 1).

However, focusing on chemical subgroups (ATC IV), inhaled anticholinergic agent (R03BB) use had a larger sex difference in the overall adjusted prevalence in the C19G (male RR, 1.44) (Supplementary Table S5). Adrenergic agent combined with corticosteroid (R03AK) use had the highest prevalence in the C19G (6.1% vs. 4.0%; RR, 1.53) among those aged 40–59 years (Supplementary Table S5). Glucocorticoid (R03BA) use had the highest prevalence in the C19G among those aged 40–59 years (10.4% vs. 7.3%; RR, 1.42) (Supplementary Table S5) and those aged 60–79 years (14.9% vs. 11.4%; RR, 1.31) (Supplementary Table S5). Higher prevalence rates of anticholinergic (R03BB) use (11.9% vs. 9.8%; RR, 1.23) and piperazine derivative (R06AE) use (7.1% vs. 5.5%; RR, 1.30) were observed in the C19G among those aged > 80 years (Supplementary Table S5).

## Discussion

The COVID-19 pandemic has imposed great challenges on healthcare systems worldwide. Some literature has been published on the clinical aspects of, possible treatments for and risk factors in patients with COVID-19^23–26^. Nevertheless, apart from a few studies, the epidemiology of and drug use profiles in patients with COVID-19 has not been studied. To our knowledge, this is the first study addressing this topic.

Most of our COVID-19 population comprised middle-aged men (55 ± 19 years; 80% were > 40 years of age) and those receiving ≥ 1 drug (74.5% of patients, including 35% with a polypharmacy regimen).

In general, our results revealed four profiles. The first comprises an age range of 0–39 (median age, 27 ± 9) years, male sex, no exposure to any drug in approximately half of the patients and a very low prevalence of polytherapy. The second comprises an age range of 40–59 (median age, 51 ± 5) years, male sex, use of 1–4 drugs in nearly half of the patients and a low prevalence of polytherapy (< 25%). The third comprises an age range of 60–79 (median age, 68 ± 6) years, male sex, use of ≥ 1 drug in 90% of the patients and polytherapy in more than half of the patients. The final profile comprises an age > 80 (median age 85 ± 4) years, female sex, use of ≥ 1 drug in 94% of the patients and polytherapy in 78% of the patients.

The analyses of drug-utilisation profiles highlighted differences between the C19G and GPG in terms of the prevalence of drug exposure. The drug categories with a difference ≥ 30% were antithrombotic agents (B01), antiepileptics (N03), anti-hyperuricaemics/anti-gout (M04) and cardiac therapy agents (C01). The higher prevalence rates associated with drugs in categories C01, B01 and M04 indicate a frequent pattern of cardiovascular and metabolic comorbidities in COVID-19 populations, as reported in other studies^4,5,8^. It is of some relevance that B01 drug use had the largest difference between the COVID-19 and general populations. This therapeutic profile indicates the presence of cardiovascular complications (including venous thromboembolism), supporting the hypothesis of an increased risk associated with COVID-19 infection in these patients^8^.

With regard to the higher prevalence of use of drugs in the M04 category, a retrospective cohort study including 131,565 patients and 252,763 controls, using data from the UK Clinical Practice Research Datalink, reported an increased risk of pneumonia (hazard ratio, 1.27; 95% CI 1.18–1.36) in patients with gout^27^.

There is no clear association between epilepsy and the risk of developing COVID-19. Nevertheless, epilepsy may be associated with other comorbidities or a component of congenital/inherited syndromes that may affect the immune system. Additionally, anti-epileptic agents can be used in association with other medications that can influence the immune system (e.g., adrenocorticotropic hormones, corticosteroids, everolimus, immunotherapy agents), and this may increase the infection risk^28^. Moreover, these patients may require frequent clinical evaluation, which may explain (at least in part) their increased risk of healthcare-associated infections.

Notably, the adjusted prevalence of the use of drugs acting on the RAS (C09) was not different between the C19G and GPG (RR, 1.02; 95% CI, 1.01–1.02). This result is in accordance with evidence from a retrospective study involving a COVID-19 cohort in Italy^29^ and supports the position of the European Society of Cardiology^30^. Furthermore, no major differences were noted for any category of antihypertensive drugs. Corroborating our results, a recent study carried out in the United States revealed no association between ACEI or ARB use and COVID-19, supporting the recommendation of continuing ACEI and ARB use in the setting of the COVID-19 pandemic^31^. This was further explored in a recent Brazilian study that confirmed that among patients hospitalised with mild to moderate COVID-19 who were taking ACEIs or ARBs before hospital admission, there were no significant differences in the mean number of days alive and out of the hospital between those who discontinued and continued these medications^32^.

Stratification by age showed a higher prevalence of use of drugs in categories B01, B03, C09 and C10 in people aged < 40 years. This evidence should be interpreted with caution because the number of such patients was very small. Nevertheless, a morbidity pattern similar to that in older patients was observed in these patients. Conversely, in COVID-19 patients aged > 60 years, there was no significant difference in the prevalence of drug use for cardiometabolic diseases compared with that in the GPG, but the prevalence rates of drug use for respiratory diseases and neurological diseases were increased in the C19G.

A large number of males took analgesics (N02) and drugs for cardiac therapy (C01). A high number of females took anti-anaemia agents (B03) and anti-epileptic agents (N03). Early descriptions of COVID-19 suggested a male preponderance^23,24,33^. Sex-based immunological, genetic, and lifestyle differences (e.g., tobacco smoking) have been postulated to explain the male preponderance of COVID-19^34^. In a population of 507 patients with COVID-19 between 13 and 31 January 2020 (including 364 from mainland China), 281 patients were male (55%), and the median age was 46 (IQR, 35–60) years^35^. Zhou and colleagues described 191 COVID-19 patients from Wuhan (Hubei Province, China) during the first month of the outbreak. That cohort had a median age of 56 (IQR, 46.0–67.0) years, with 62% being male and 48% with comorbidities^23^. Additionally, data from Italy revealed a higher prevalence of COVID-19 in males than in females^36,37^. However, sex- and age-disaggregated data revealed the opposite to be true for women aged > 80 years in Campania. National data from Italy revealed that in those aged 20–29 years, 56.5% of the diagnosed patients were female, and only after the age of 50 years does the male preponderance of COVID-19 increase. Thus, the male preponderance of COVID-19 should be interpreted with caution because sex-disaggregated data are incomplete, and more robust evidence is needed.

Our study was not designed to define the association between drug use, comorbidities, risk of adverse outcomes and outcomes in COVID-19 patients. The associations between the use of certain drugs and susceptibility to SARS-CoV-2 infection (e.g., predictive factors for poor outcome) must be studied in a large cohort with a control group and robust clinical data. This was a retrospective study of health records. Additional detailed patient information (mainly regarding clinical outcomes) was not available at the time of the analysis. Despite these limitations, we delineated the drug use profiles and epidemiological and demographic characteristics of 1532 Italian patients with COVID-19. This information provides the first evidence of the association between drug utilisation and COVID-19 risk, giving us a solid background for further analyses and interpretations using new data.

## Conclusions

In conclusion, the current data provide baseline information about the complexity of patients affected by COVID-19, showing frequencies and differences in drug utilisation profiles in COVID-19 patients compared with the general population. The higher prevalence rates of C01, B01 and M04 use were probably linked to related comorbidities (i.e., cardiovascular, metabolic). Nevertheless, the prevalence of the use of drugs acting on RAS, such as other anti-hypertensive drugs, did not show a higher prevalence among COVID-19 patients than among the general population. Since these pilot data were derived from the first month of documented COVID-19 cases in the Campania region (southern Italy), our results highlight the need for further case–control studies to define the effects of medications and comorbidities on susceptibility to and associated mortality from COVID-19 infection. Finally, to better understand the global epidemiology of COVID-19, reproducible and comparable results from cohorts from multiple countries and regions are needed for further investigation and meta-analysis.

## Supplementary Information

Supplementary Information.
