# Supplementary material for: Drug-utilisation profiles and COVID-19
Source: Sci Rep. 2021 Apr 26;11:8913. doi: 10.1038/s41598-021-88398-y (PMC8076316; doi:10.1038/s41598-021-88398-y)
Supplement: Supplementary file 1 — Supplementary Information. [file 41598_2021_88398_MOESM1_ESM.pdf]

Supplementary information

Drug-utilisation Profiles and COVID-19

Valentina Orlando[1,*], Enrico Coscioni[2], Ilaria Guarino[1], Sara Mucherino[1], Alessandro Perrella[3], Ugo Trama[4], Giuseppe Limongelli[5,+] and Enrica Menditto[1,+,*]

[1]CIRFF, Center of Drug Utilisation and Pharmacoeconomics, University of Naples Federico II, Naples, 80131, Italy
[2]Division of Cardiac Surgery, AOU San Giovanni di Dio e Ruggi d'Aragona, Salerno, 84131, Italy
[3]Infectious Disease of Healthcare Direction, AORN Antonio Cardarelli, Naples, 80131, Italy
[4]Regional Pharmaceutical Unit, Campania Region, Naples, 80143, Italy
[5]Department of Translational Medical Science, University of Campania Luigi Vanvitelli, Monaldi Hospital, Naples, 80131, Italy

[+]These authors contributed equally to this work
*valentina.orlando@unina.it, enrica.menditto@unina.it

Characteristics	
Geographic area	Campania, Italy
Population covered	~5.8 million inhabitants
Age span covered	Whole population
Time span covered [b]	2010–2019
Scope	Drug utilisation and outcome research; real-world evidences for public health; pharmacoepidemiologic and pharmacoeconomic analysis
Data sources	Specific fields in the data sources contributing to a database
Demographic information	Patient ID; sex; date of birth; municipality of residence; district; local health unit
Outpatient pharmacy records	Patient ID; drug code; prescription date; delivery date; quantity; ATC code; price; defined daily dose; drug distribution channel (file F, file D)
Hospital-discharge records	Patient ID; type of admission; date of admission; reasons for discharge; diagnoses (ICD-9 code); procedures (ICD-9 code); date of discharge; disease-related group
Type of codes for diagnoses	Hospital: ICD-9-CM
Type of codes for medications	ATC classification

ATC = Anatomical Therapeutic Chemical; ICD-9-CM = *International Classification of Diseases*, 9th Revision, Clinical Modification.
[a] For this study, outpatient pharmacy records were used to identify medication use.
[b] Time span covered was 2009–2018 for hospital-discharge records and 2014–2019 for outpatient pharmacy records.

Supplementary Table S1. CaReDB characteristics[a].

ATC II	Therapeutic subgroup	Prevalence of drug use (%)															
		0 - 39 years				40 - 59 years				60 - 79 years				≥ 80 years			
		Unadjusted		Adjusted		Unadjusted		Adjusted		Unadjusted		Adjusted		Unadjusted		Adjusted	
		C19G	GPG	C19G	GPG	C19G	GPG	C19G	GPG	C19G	GPG	C19G	GPG	C19G	GPG	C19G	GPG
A02	Acid-related Disorders	9.1	6.8	9.6	6.8	31.6	26.5	32.4	26.5	56.2	56.5	57.7	56.5	74.7	75.9	75.2	75.8
A07	Antidiarrheals, Intestinal Antiinflammatory/ Antiinfective Agents	4.7	2.6	4.7	2.6	7.1	6.2	6.9	6.2	14.4	13.4	14.8	13.4	14.4	16.9	14.4	16.9
A10	Drugs used in diabetes	< 0.1	0.4	< 0.1	0.4	4.5	3.7	4.5	3.7	18.5	17.2	17.3	17.2	26.0	22.0	25.4	22.0
A11	Vitamins	2.5	2.4	2.6	2.3	12.3	13.1	14.6	13.0	25.9	31.7	33.1	31.6	28.8	39.2	30.0	39.1
B01	Antithrombotic agents	2.8	1.1	2.9	1.1	10.7	5.7	10.5	5.7	37.9	27.1	36.8	27.1	59.6	48.3	59.8	48.2
B03	Anti-anemia Agents	3.8	2.5	4.4	2.5	5.2	4.2	5.7	4.2	5.8	7.1	6.4	7.1	16.4	16.5	17.1	16.5
C01	Cardiac therapy	< 0.1	0.1	< 0.1	0.1	1.4	0.7	1.4	0.7	8.8	5.5	8.2	5.5	15.1	16.5	14.8	16.5
C03	Diuretics	0.3	0.2	0.4	0.2	2.4	2.2	2.4	2.2	11.9	11.4	13.0	11.4	32.9	32.4	33.3	32.4
C07	Beta-Blockers	0.6	0.6	0.6	0.6	11.9	10.7	11.8	10.7	30.5	32.1	29.8	32.1	42.5	43.8	42.5	43.8
C08	Calcium channel blockers	0.3	0.2	0.3	0.2	4.9	3.8	4.4	3.8	18.1	15.4	17.3	15.4	26.0	24.8	26.0	24.8
C09	Drugs Acting on the Renin–Angiotensin System (RAS)	1.3	0.9	1.1	0.9	22.4	18.6	21.6	18.7	55.6	55.9	54.7	55.9	64.4	71.2	65.6	71.2
C10	Lipid modifying agents	0.6	0.5	0.5	0.5	12.1	10.4	11.9	10.4	36.4	39.4	36.2	39.3	34.9	42.7	34.6	42.7
H02	Corticosteroids for systemic use	9.4	10.6	9.8	10.6	14.6	15.1	15.2	15.1	20.2	21.1	20.9	21.1	28.8	24.3	28.7	24.3
H03	Thyroid therapy	0.6	1.1	0.8	1.1	4.2	4.5	4.7	4.5	6.8	8.2	8.9	8.2	7.5	8.2	7.9	8.2
J01	Antibacterial Drugs for Systemic Use	35.1	35.2	35.4	35.1	43.6	43.8	45.4	43.7	61.1	61.5	62.6	61.4	70.5	67.8	70.8	67.8
J02	Antimycotics for systemic use	2.5	1.9	2.7	1.9	3.8	3.1	3.7	3.1	2.7	3.0	2.8	3.0	2.7	2.9	2.9	2.9
M01	Anti-inflammatory and Antirheumatic Drugs	8.5	5.2	9.1	5.2	21.5	24.9	22.6	24.8	34.4	42.9	35.2	42.9	40.4	43.0	40.0	43.0
M04	Anti-gout Preparations	< 0.1	0.1	< 0.1	0.1	3.3	1.2	3.2	1.2	9.7	7.3	9.4	7.3	19.2	17.9	19.2	17.9
N02	Analgesic Drugs	1.3	0.7	1.3	0.7	4.7	3.8	4.5	3.7	8.6	8.8	9.7	8.8	15.1	14.0	15.4	13.9
N03	Antiepileptics	1.3	1.2	1.3	1.2	4.7	3.4	5.1	3.4	7.8	6.5	8.6	6.5	13.7	9.4	14.6	9.4
N06	Psychoanaleptics	0.9	1.1	1.0	1.1	4.5	5.3	4.9	5.2	9.5	10.0	10.5	10.0	26.0	17.8	27.3	17.7
R03	Drugs for obstructive airway diseases	10.3	14.4	10.2	14.4	15.9	12.2	16.7	12.2	23.5	21.4	24.4	21.4	32.9	28.3	32.3	28.3
R06	Antihistamines for systemic use	8.5	7.7	8.5	7.7	8.1	8.4	9.0	8.4	9.7	11.0	10.5	11.0	11.0	9.8	11.5	9.8

Supplementary Table S2. Differences in Prevalence of drug use between C19G and GPG by Therapeutic subgroup (ATC II).

ATC II	Therapeutic subgroup	Adjusted RR C19G/GPG (95%CI)						
		Overall	Gender		Age Groups			
			Male	Female	0 - 39 years	40 - 59 years	60 - 79 years	≥ 80 years
**A02**	**Acid-related Disorders**	1.12 (1.11–1.12)	1.05 (1.05–1.05)	1.17 (1.17–1.17)	1.45 (1.45–1.46)	1.22 (1.22–1.23)	1.02 (1.02–1.02)	1.01 (1.01–1.02)
**A07**	**Antidiarrheals, Intestinal Antiinflammatory/ Antiinfective Agents**	1.17 (1.16–1.17)	1.22 (1.22–1.23)	1.13 (1.12–1.13)	1.83 (1.82–1.84)	1.11 (1.10–1.12)	1.11 (1.11–1.12)	0.85 (0.84–0.86)
**A10**	**Drugs used in diabetes**	1.07 (1.06–1.06)	1.07 (1.07–1.08)	1.06 (1.05–1.07)	-	1.24 (1.23–1.25)	1.02 (1.02–1.03)	1.15 (1.14–1.16)
**A11**	**Vitamins**	1.02 (1.01–1.02)	1.08 (1.07–1.08)	1.01 (1.00–1.01)	1.10 (1.09–1.11)	1.13 (1.12–1.13)	1.05 (1.04–1.05)	0.77 (0.76–0.77)
**B01**	**Antithrombotic agents**	1.47 (1.46–1.47)	1.50 (1.50–1.51)	1.44 (1.44–1.45)	2.91 (2.88–2.95)	1.82 (1.81–1.84)	1.36 (1.36–1.37)	1.30 (1.29–1.31)
**B03**	**Anti-anemia Agents**	1.28 (1.27–1.28)	1.02 (1.01–1.03)	1.39 (1.38–1.40)	1.74 (1.73–1.75)	1.38 (1.37–1.39)	1.06 (1.05–1.06)	1.08 (1.07–1.09)
**C01**	**Cardiac therapy**	1.32 (1.31–1.33)	1.47 (1.46–1.48)	1.20 (1.19–1.21)	4.63 (4.40–4.87)	2.09 (2.05–2.13)	1.50 (1.49–1.51)	0.94 (0.94–0.95)
**C03**	**Diuretics**	1.12 (1.11–1.12)	0.95 (0.95–0.96)	1.23 (1.23–1.24)	2.27 (2.21–2.34)	1.09 (1.08–1.10)	1.14 (1.14–1.15)	1.08 (1.07–1.08)
**C07**	**Beta-Blockers**	0.98 (0.98–0.98)	1.09 (1.09–1.09)	0.90 (0.90–0.91)	1.07 (1.05–1.09)	1.10 (1.10–1.11)	0.93 (0.93–0.94)	0.97 (0.97–0.98)
**C08**	**Calcium channel blockers**	1.11 (1.10–1.11)	1.23 (1.23–1.24)	1.00 (0.99–1.00)	1.39 (1.36–1.43)	1.14 (1.14–1.15)	1.13 (1.12–1.13)	1.05 (1.04–1.05)
**C09**	**Drugs Acting on the Renin–Angiotensin System (RAS)**	1.02 (1.01–1.01)	1.05 (1.04–1.05)	0.99 (0.99–0.99)	1.27 (1.26–1.29)	1.16 (1.15–1.16)	0.98 (0.98–0.98)	0.93 (0.93–0.94)
**C10**	**Lipid modifying agents**	0.95 (0.95–0.95)	0.96 (0.96–0.97)	0.94 (0.94–0.95)	1.13 (1.11–1.15)	1.15 (1.14–1.15)	0.93 (0.93–0.93)	0.81 (0.81–0.81)
**H02**	**Corticosteroids for systemic use**	1.00 (1.00–1.00)	0.93 (0.93–0.94)	1.06 (1.05–1.06)	0.92 (0.92–0.93)	1.01 (1.01–1.01)	0.99 (0.99–1.00)	1.21 (1.20–1.22)
**H03**	**Thyroid therapy**	1.02 (1.02–1.02)	0.97 (0.96–0.98)	1.04 (1.03–1.04)	0.71 (0.70–0.72)	1.06 (1.05–1.06)	1.09 (1.09–1.10)	0.96 (0.95–0.98)
**J01**	**Antibacterial Drugs for Systemic Use**	1.02 (1.02–1.02)	1.00 (1.00–1.00)	1.05 (1.04–1.05)	1.01 (1.00–1.01)	1.04 (1.03–1.04)	1.02 (1.02–1.02)	1.04 (1.04–1.05)
**J02**	**Antimycotics for systemic use**	1.17 (1.16–1.17)	1.41 (1.40–1.43)	1.03 (1.03–1.04)	1.43 (1.42–1.44)	1.18 (1.17–1.19)	0.91 (0.90–0.92)	0.98 (0.96–1.00)
**M01**	**Anti-inflammatory and Antirheumatic Drugs**	0.95 (0.95–0.95)	0.92 (0.91–0.92)	0.98 (0.98–0.98)	1.74 (1.73–1.75)	0.91 (0.91–0.91)	0.82 (0.82–0.82)	0.93 (0.93–0.94)
**M04**	**Anti-gout Preparations**	1.37 (1.36–1.37)	1.20 (1.19–1.21)	1.55 (1.54–1.56)	-	2.63 (2.60–2.67)	1.30 (1.30–1.31)	1.12 (1.11–1.13)
**N02**	**Analgesic Drugs**	1.20 (1.19–1.20)	1.41 (1.40–1.42)	1.09 (1.09–1.10)	1.94 (1.91–1.97)	1.27 (1.26–1.28)	1.11 (1.10–1.11)	1.10 (1.09–1.11)
**N03**	**Antiepileptics**	1.39 (1.38–1.40)	0.99 (0.99–1.00)	1.69 (1.68–1.70)	1.11 (1.10–1.12)	1.52 (1.51–1.53)	1.32 (1.31–1.33)	1.55 (1.53–1.57)
**N06**	**Psychoanaleptics**	1.12 (1.11–1.12)	1.00 (0.99–1.00)	1.18 (1.17–1.18)	0.93 (0.92–0.94)	0.93 (0.92–0.94)	1.05 (1.04–1.06)	1.54 (1.53–1.55)
**R03**	**Drugs for obstructive airway diseases**	1.05 (1.04–1.04)	1.00 (1.00–1.01)	1.08 (1.08–1.08)	0.71 (0.71–0.71)	1.37 (1.37–1.38)	1.15 (1.15–1.16)	1.14 (1.13–1.15)
**R06**	**Antihistamines for systemic use**	1.06 (1.05–1.06)	0.95 (0.94–0.95)	1.14 (1.14–1.15)	1.10 (1.09–1.10)	1.07 (1.07–1.08)	0.96 (0.96–0.97)	1.18 (1.17–1.19)

Supplementary Table S3. Gender and Age group differences in drug use between C19G and GPG by Pharmacological group (ATC II).

ATC IV	Chemical subgroup	Prevalence of drug use (%)															
		0 - 39 years				40 - 59 years				60 - 79 years				≥ 80 years			
		Unadjusted		Adjusted		Unadjusted		Adjusted		Unadjusted		Adjusted		Unadjusted		Adjusted	
		C19G	GPG	C19G	GPG	C19G	GPG	C19G	GPG	C19G	GPG	C19G	GPG	C19G	GPG	C19G	GPG
A02AD	Aluminium. calcium and magnesium	0.9	1.0	0.9	1.0	4.7	3.6	5.0	3.6	6.2	6.4	5.9	6.4	6.2	6.1	6.9	6.1
A02BC	Proton pump inhibitors	6.3	5.2	6.8	5.2	29.5	22.8	30.1	22.8	53.1	52.7	54.5	52.7	74.0	73.6	74.4	73.5
A02BX	Other drugs for peptic ulcers	2.8	1.8	3.0	1.8	6.3	6.2	6.4	6.1	9.9	10.2	10.8	10.2	7.5	9.4	7.5	9.4
A07AA	Antibiotics	2.8	2.2	2.8	2.2	4.9	4.8	5.0	4.8	10.7	11.3	11.4	11.3	13.0	15.3	13.3	15.2
A10BA	Biguanides	< 0.1	0.2	< 0.1	0.2	3.4	2.7	3.4	2.7	12.6	11.6	11.5	11.6	15.1	10.7	14.6	10.7
A11CC	Vitamin D and analogues	2.5	2.3	2.6	2.3	12.3	13.1	14.6	13.0	25.9	31.7	33.1	31.6	28.8	39.2	30.0	39.1
B01AB	Heparin group	2.2	0.8	2.3	0.8	3.6	1.8	4.1	1.8	7.2	4.6	7.1	4.6	11.0	8.2	10.6	8.2
B01AC	Platelet aggregation inhibitors	0.6	0.3	0.5	0.3	6.7	4.0	6.1	4.0	30.7	23.3	29.7	23.3	45.2	40.8	46.1	40.8
B03BB	Folic acid and derivatives	2.8	1.5	3.3	1.5	3.3	2.4	3.2	2.4	3.9	4.4	4.1	4.4	9.6	9.6	9.8	9.6
C03CA	Sulfonamides	0.3	0.1	0.4	0.1	0.9	1.5	0.9	1.5	8.8	8.7	9.8	8.7	27.4	27.5	27.7	27.5
C07AB	β blocking agents. selective	0.3	0.4	0.3	0.4	8.9	8.0	8.6	8.0	24.7	24.9	24.1	24.9	34.2	34.5	33.7	34.5
C08CA	Dihydropyridine derivatives	0.3	0.2	0.3	0.2	4.7	3.7	4.3	3.7	16.9	14.6	16.5	14.6	22.6	22.8	22.7	22.7
C09AA	ACE inhibitors	0.3	0.3	0.3	0.3	5.6	5.5	5.3	5.5	14.8	15.3	13.6	15.3	18.5	20.9	18.3	20.9
C09BA	ACE inhibitors and diuretics	< 0.1	0.1	< 0.1	0.1	2.9	2.7	3.0	2.7	9.1	9.0	9.1	9.0	8.9	11.9	9.2	11.9
C09CA	Angiotensin II receptor blockers	0.6	0.2	0.5	0.2	6.3	5.0	6.0	5.0	16.7	15.4	16.0	15.4	22.6	21.2	24.2	21.2
C09DA	Angiotensin II receptor blockers and diuretics	0.3	0.1	0.3	0.1	5.2	3.8	5.0	3.8	15.2	14.9	16.7	14.9	16.4	20.0	16.5	20.0
C10AA	HMG CoA reductase inhibitors	0.6	0.3	0.5	0.3	9.4	8.4	9.2	8.4	31.3	34.6	31.1	34.5	30.8	38.9	30.4	38.9
H02AB	Glucocorticoids	9.4	10.6	9.8	10.6	14.6	15.1	15.2	15.1	20.2	21.1	20.9	21.1	28.8	24.3	28.7	24.3
H03AA	Thyroid hormones	0.6	1.0	0.8	1.0	4.2	4.3	4.7	4.3	5.6	7.5	7.9	7.5	6.8	6.5	7.1	6.5
J01CA	Penicillins with extended spectrum	2.8	3.1	2.6	3.1	3.4	3.8	3.6	3.8	4.5	6.0	4.7	6.0	3.4	5.0	3.5	5.0
J01CR	Combinations of penicillins	16.3	17.1	16.2	17.0	19.7	21.6	20.2	21.5	29.0	29.1	29.6	29.0	26.7	27.4	27.3	27.4
J01DD	Third-generation cephalosporins	11.6	9.8	11.8	9.8	12.1	11.9	12.5	11.9	19.1	20.0	19.1	20.0	38.4	29.1	37.3	29.1

**J01FA**	**Macrolides**	10.3	11.0	10.5	11.0	15.0	11.9	16.2	11.9	16.1	16.5	16.6	16.5	12.3	16.5	12.9	16.5
**J01MA**	**Fluoroquinolones**	3.4	3.2	3.7	3.2	12.7	10.2	13.1	10.2	19.5	20.9	19.7	20.9	28.8	28.2	29.0	28.2
**J01XX**	**Other antibacterials**	1.6	1.9	1.8	1.9	5.6	4.6	6.7	4.5	7.2	8.3	9.0	8.3	8.9	13.0	9.0	12.9
**J02AC**	**Antimycotics For Systemic Use**	2.5	1.9	2.7	1.9	3.8	3.1	3.7	3.1	2.7	3.0	2.8	3.0	2.7	2.9	2.9	2.9
**M01AB**	**Acetic acid derivatives and related substance**	3.4	1.7	3.5	1.7	8.5	9.8	8.7	9.8	16.1	18.4	16.9	18.4	17.1	18.3	17.1	18.3
**M01AE**	**Propionic acid derivatives**	4.7	3.0	5.2	3.0	9.8	13.3	10.5	13.2	18.1	22.0	18.3	22.0	18.5	21.5	18.5	21.5
**M01AH**	**Coxibs**	0.3	0.3	0.3	0.3	2.7	3.3	3.0	3.3	6.6	8.1	7.5	8.1	8.9	8.0	8.8	8.0
**M01AX**	**Other antiinflammatory and antirheumatic agents non-steroidal anti-inflammatory drugs**	0.9	0.9	1.0	0.8	2.9	5.0	2.6	4.9	5.6	9.7	5.2	9.7	8.9	9.2	8.5	9.2
**M04AA**	**Preparations inhibiting uric acid production**	< 0.1	0.1	< 0.1	0.1	2.9	1.2	2.8	1.2	9.1	7.1	8.5	7.1	19.2	17.7	19.2	17.7
**N03AX**	**Other antiepileptics**	0.6	0.6	0.6	0.6	2.9	2.2	3.4	2.2	5.3	5.1	5.5	5.1	11.0	7.2	11.7	7.2
**N06AB**	**Selective serotonin reuptake inhibitors**	0.3	0.8	0.4	0.8	3.1	3.8	3.3	3.8	4.7	6.9	5.4	6.9	11.6	11.1	12.5	11.1
**N06AX**	**Other antidepressants**	0.6	0.3	0.6	0.3	1.4	1.6	1.5	1.6	5.1	3.6	5.2	3.6	15.1	7.9	15.9	7.9
**R03AK**	**Adrenergics in combination with corticosteroids**	1.6	1.8	1.6	1.8	5.8	4.0	6.1	4.0	7.6	8.1	7.2	8.1	10.3	10.9	9.8	10.9
**R03BA**	**Glucocorticoids**	8.2	11.5	8.1	11.4	9.6	7.3	10.4	7.3	14.0	11.4	14.9	11.4	13.7	14.1	13.7	14.1
**R03BB**	**Anticholinergics**	0.3	0.2	0.3	0.2	1.3	1.0	1.1	1.0	6.8	5.2	6.6	5.2	13.0	9.7	11.9	9.8
**R06AE**	**Piperazine derivatives**	4.7	4.3	4.7	4.3	4.3	3.9	4.8	3.9	4.9	5.8	5.3	5.8	6.8	5.5	7.1	5.5
**R06AX**	**Other antihistamines for systemic use**	4.1	3.8	4.1	3.8	4.2	5.0	4.7	4.9	5.6	5.9	6.3	5.9	4.1	5.0	4.4	5.0

Supplementary Table S4. Differences in Prevalence of drug use between C19G and GPG by Chemical Subgroup (ATC IV).

ATC IV	Chemical subgroup	Adjusted RR C19G/GPG (95%CI)						
		Overall	Gender		Age Groups			
			Male	Female	0 - 39 years	40 - 59 years	60 - 79 years	≥ 80 years
A02AD	Aluminium, calcium and magnesium	1.10 (1.09–1.11)	1.14 (1.13–1.15)	1.08 (1.07–1.09)	0.92 (0.91–0.93)	1.39 (1.37–1.40)	0.92 (0.92–0.93)	1.14 (1.12–1.15)
A02BC	Proton pump inhibitors	1.14 (1.13–1.14)	1.06 (1.06–1.06)	1.20 (1.19–1.20)	1.36 (1.35–1.37)	1.32 (1.32–1.33)	1.03 (1.03–1.04)	1.03 (1.03–1.04)
A02BX	Other drugs for peptic ulcers	1.10 (1.09–1.11)	1.06 (1.06–1.07)	1.13 (1.12–1.13)	1.69 (1.67–1.70)	1.05 (1.04–1.05)	1.06 (1.06–1.06)	0.80 (0.79–0.80)
A07AA	Antibiotics	1.03 (1.02–1.03)	1.06 (1.05–1.06)	1.01 (1.01–1.02)	1.28 (1.27–1.29)	1.04 (1.03–1.04)	1.01 (1.01–1.02)	0.87 (0.86–0.88)
A10BA	Biguanides	1.09 (1.08–1.09)	1.16 (1.15–1.17)	1.01 (1.00–1.01)	-	1.24 (1.22–1.25)	0.98 (0.98–0.99)	1.36 (1.35–1.38)
A11CC	Vitamin D and analogues	1.02 (1.01–1.02)	1.08 (1.07–1.09)	1.01 (1.00–1.01)	1.10 (1.09–1.11)	1.13 (1.12–1.13)	1.05 (1.05–1.05)	0.77 (0.76–0.77)
B01AB	Heparin group	1.88 (1.87–1.89)	1.93 (1.92–1.95)	1.85 (1.84–1.87)	3.19 (3.15–3.23)	2.27 (2.24–2.29)	1.55 (1.55–1.56)	1.38 (1.37–1.40)
B01AC	Platelet aggregation inhibitors	1.29 (1.28–1.29)	1.32 (1.32–1.33)	1.26 (1.26–1.27)	1.94 (1.89–1.98)	1.52 (1.51–1.53)	1.28 (1.28–1.29)	1.18 (1.18–1.19)
B03BB	Folic acid and derivatives	1.31 (1.30–1.32)	1.27 (1.26–1.28)	1.33 (1.32–1.33)	2.22 (2.19–2.24)	1.36 (1.35–1.38)	0.93 (0.93–0.93)	1.02 (1.01–1.03)
C03CA	Sulfonamides	1.07 (1.06–1.07)	0.86 (0.86–0.87)	1.20 (1.19–1.21)	-	0.59 (0.59–0.60)	1.13 (1.13–1.14)	1.06 (1.06–1.07)
C07AB	β blocking agents, selective	0.99 (0.98–0.99)	1.15 (1.14–1.15)	0.87 (0.87–0.87)	-	1.07 (1.06–1.07)	0.98 (0.98–0.98)	0.98 (0.97–0.98)
C08CA	Dihydropyridine derivatives	1.11 (1.10–1.11)	1.19 (1.18–1.19)	1.03 (1.03–1.04)	-	1.16 (1.15–1.16)	1.14 (1.14–1.15)	1.00 (0.99–1.00)
C09AA	ACE inhibitors	0.91 (0.90–0.91)	0.96 (0.96–0.97)	0.84 (0.84–0.85)	-	0.97 (0.96–0.97)	0.89 (0.89–0.90)	0.87 (0.87–0.88)
C09BA	ACE inhibitors and diuretics	0.97 (0.96–0.97)	0.95 (0.94–0.96)	0.98 (0.97–0.98)	-	1.09 (1.08–1.10)	1.02 (1.02–1.02)	0.78 (0.77–0.78)
C09CA	Angiotensin II receptor blockers	1.13 (1.12–1.14)	1.16 (1.15–1.16)	1.11 (1.11–1.12)	2.33 (2.27–2.39)	1.20 (1.19–1.21)	1.05 (1.05–1.05)	1.18 (1.17–1.19)
C09DA	Angiotensin II receptor blockers and diuretics	1.10 (1.09–1.11)	1.13 (1.13–1.14)	1.08 (1.08–1.09)	-	1.34 (1.33–1.35)	1.12 (1.12–1.12)	0.83 (0.82–0.83)
C10AA	HMG CoA reductase inhibitors	0.92 (0.92–0.93)	0.97 (0.96–0.97)	0.89 (0.88–0.89)	1.78 (1.74–1.82)	1.09 (1.09–1.10)	0.91 (0.91–0.91)	0.78 (0.78–0.79)
H02AB	Glucocorticoids	1.00 (1.00–1.01)	0.93 (0.93–0.94)	1.06 (1.05–1.06)	0.92 (0.92–0.93)	1.01 (1.01–1.01)	0.99 (0.99–1.00)	1.21 (1.20–1.22)
H03AA	Thyroid hormones	1.05 (1.04–1.05)	0.89 (0.88–0.90)	1.08 (1.08–1.09)	0.75 (0.74–0.76)	1.11 (1.10–1.12)	1.06 (1.06–1.06)	1.09 (1.08–1.11)

**J01CA**	**Penicillins with extended spectrum**	0.83 (0.83–0.84)	0.97 (0.96–0.97)	0.73 (0.73–0.74)	0.84 (0.84–0.85)	0.94 (0.93–0.94)	0.77 (0.77–0.78)	0.69 (0.68–0.70)
**J01CR**	**Combinations of penicillins**	0.97 (0.97–0.97)	0.96 (0.96–0.97)	0.98 (0.98–0.98)	0.95 (0.95–0.95)	0.94 (0.94–0.94)	1.02 (1.02–1.02)	1.00 (0.99–1.00)
**J01DD**	**Third-generation cephalosporins**	1.10 (1.09–1.10)	1.09 (1.08–1.09)	1.11 (1.11–1.11)	1.20 (1.20–1.21)	1.05 (1.05–1.06)	0.96 (0.96–0.96)	1.28 (1.27–1.29)
**J01FA**	**Macrolides**	1.07 (1.06–1.07)	0.94 (0.94–0.95)	1.17 (1.17–1.17)	0.95 (0.95–0.96)	1.37 (1.36–1.37)	1.01 (1.01–1.01)	0.78 (0.77–0.79)
**J01MA**	**Fluoroquinolones**	1.09 (1.08–1.09)	0.99 (0.98–0.99)	1.16 (1.16–1.17)	1.17 (1.16–1.17)	1.29 (1.28–1.29)	0.95 (0.95–0.95)	1.03 (1.02–1.04)
**J01XX**	**Other antibacterials**	1.11 (1.10–1.11)	1.15 (1.14–1.16)	1.10 (1.09–1.10)	0.94 (0.93–0.95)	1.48 (1.47–1.49)	1.09 (1.09–1.10)	0.70 (0.69–0.71)
**J02AC**	**Antimycotics for systemic use**	1.17 (1.16–1.17)	1.41 (1.40–1.43)	1.03 (1.03–1.04)	1.43 (1.42–1.44)	1.18 (1.17–1.19)	0.91 (0.91–0.92)	0.98 (0.96–1.00)
**M01AB**	**Acetic acid derivatives and related sub**	1.00 (0.99–1.00)	1.04 (1.04–1.05)	0.97 (0.96–0.97)	2.07 (2.05–2.09)	0.89 (0.88–0.89)	0.92 (0.92–0.92)	0.94 (0.93–0.94)
**M01AE**	**Propionic acid derivatives**	0.92 (0.91–0.92)	0.82 (0.81–0.82)	0.98 (0.98–0.99)	1.75 (1.73–1.76)	0.79 (0.79–0.80)	0.83 (0.83–0.83)	0.86 (0.85–0.87)
**M01AH**	**Coxibs**	0.94 (0.93–0.95)	0.99 (0.98–0.99)	0.92 (0.92–0.93)	-	0.90 (0.89–0.90)	0.93 (0.93–0.93)	1.10 (1.09–1.12)
**M01AX**	**Other antiinflammatory and antirheumatic non-steroids, non-steroidal anti-inflammatory drugs**	0.63 (0.63–0.64)	0.91 (0.91–0.92)	0.45 (0.45–0.45)	1.20 (1.19–1.22)	0.53 (0.52–0.53)	0.53 (0.53–0.53)	0.92 (0.91–0.93)
**M04AA**	**Preparations inhibiting uric acid production**	1.29 (1.28–1.30)	1.22 (1.22–1.23)	1.37 (1.36–1.38)	-	2.36 (2.33–2.40)	1.21 (1.21–1.22)	1.13 (1.12–1.14)
**N03AX**	**Other antiepileptics**	1.30 (1.29–1.31)	0.91 (0.90–0.92)	1.55 (1.54–1.56)	1.03 (1.01–1.04)	1.53 (1.51–1.54)	1.08 (1.08–1.09)	1.62 (1.60–1.64)
**N06AB**	**Selective serotonin reuptake inhibitors**	0.86 (0.85–0.86)	0.67 (0.66–0.67)	0.95 (0.94–0.95)	-	0.87 (0.86–0.88)	0.79 (0.79–0.80)	1.13 (1.12–1.14)
**N06AX**	**Other antidepressants**	1.54 (1.53–1.55)	1.46 (1.45–1.48)	1.58 (1.57–1.59)	2.34 (2.29–2.39)	0.96 (0.95–0.97)	1.44 (1.44–1.45)	2.02 (2.00–2.05)
**R03AK**	**Adrenergics in combination with corticosteroids**	1.06 (1.05–1.07)	1.11 (1.11–1.12)	1.02 (1.01–1.02)	0.88 (0.87–0.89)	1.53 (1.52–1.54)	0.89 (0.89–0.89)	0.90 (0.89–0.91)
**R03BA**	**Glucocorticoids**	1.03 (1.03–1.04)	0.95 (0.95–0.95)	1.10 (1.10–1.11)	0.71 (0.70–0.71)	1.42 (1.41–1.43)	1.31 (1.31–1.32)	0.97 (0.96–0.97)
**R03BB**	**Anticholinergics**	1.25 (1.24–1.26)	1.44 (1.43–1.45)	1.03 (1.02–1.04)	-	1.10 (1.08–1.12)	1.29 (1.29–1.30)	1.23 (1.21–1.24)
**R06AE**	**Piperazine derivatives**	1.10 (1.09–1.10)	1.00 (0.99–1.00)	1.17 (1.16–1.18)	1.10 (1.09–1.11)	1.23 (1.22–1.24)	0.90 (0.90–0.91)	1.30 (1.28–1.32)
**R06AX**	**Other antihistamines for systemic use**	1.02 (1.01–1.02)	0.82 (0.81–0.82)	1.17 (1.16–1.17)	1.09 (1.08–1.09)	0.94 (0.94–0.95)	1.07 (1.07–1.08)	0.88 (0.87–0.89)

Supplementary Table S5. Gender and Age group differences in drug use between C19G and GPG by Chemical Pharmacological Subgroup (ATC IV)
